# Supplementary material for: The Systems Biology Research Tool: evolvable open-source software
Source: BMC Syst Biol. 2008 Jun 29;2:55. doi: 10.1186/1752-0509-2-55 (PMC2446383; doi:10.1186/1752-0509-2-55)
Supplement: Additional file 1 — SBRT Archive. An archive of the current version of the Systems Biology Research Tool. [file 1752-0509-2-55-S1.zip › sbrt-1.4.0/doc/developers_guide/api/sbrt/kernel/math/VectorComparer.html]

VectorComparer


|  |  |  |
| --- | --- | --- |
| **Overview** \| **Package** \| **Class** \| **Use** \| **Tree** \| **Deprecated** \| **Index** \| **Help** | |  |
| **PREV CLASS**   **NEXT CLASS** | **FRAMES**    **NO FRAMES**     **All Classes** |  |
| SUMMARY: NESTED \| FIELD \| CONSTR \| METHOD | DETAIL: FIELD \| CONSTR \| METHOD |  |


---


## sbrt.kernel.math Class VectorComparer

```
java.lang.Object
  sbrt.kernel.math.VectorComparer
```

---

``` public class VectorComparer extends java.lang.Object ```

This class is used to compare vectors of double precision numbers.

**Author:**
:   This class was written and documented by Jeremiah Wright while in the
    Wagner lab.

---

| **Nested Class Summary** | |
| --- | --- |
| `static class` | `VectorComparer.Diff<V>`             Instances of this class are used to hold a variable and a pair of corresponding intervals. |


**Constructor Summary**

`VectorComparer()`


| **Method Summary** | |
| --- | --- |
| `boolean` | `areSame(java.util.Map<?,java.lang.Double> m1, java.util.Map<?,java.lang.Double> m2, double tolerance)` |
| `<V> java.util.Set<VectorComparer.Diff<V>>` | `getDiffs(java.util.Map<? extends V,java.lang.Double> m1, java.util.Map<? extends V,java.lang.Double> m2, double tolerance)` |

**Methods inherited from class java.lang.Object**

`clone, equals, finalize, getClass, hashCode, notify, notifyAll, toString, wait, wait, wait`

**Constructor Detail**

### VectorComparer

```
public VectorComparer()
```


**Method Detail**

### areSame

```
public boolean areSame(java.util.Map<?,java.lang.Double> m1,
                       java.util.Map<?,java.lang.Double> m2,
                       double tolerance)
```

---


### getDiffs

```
public <V> java.util.Set<VectorComparer.Diff<V>> getDiffs(java.util.Map<? extends V,java.lang.Double> m1,
                                                          java.util.Map<? extends V,java.lang.Double> m2,
                                                          double tolerance)
```


---


|  |  |  |
| --- | --- | --- |
| **Overview** \| **Package** \| **Class** \| **Use** \| **Tree** \| **Deprecated** \| **Index** \| **Help** | |  |
| **PREV CLASS**   **NEXT CLASS** | **FRAMES**    **NO FRAMES**     **All Classes** |  |
| SUMMARY: NESTED \| FIELD \| CONSTR \| METHOD | DETAIL: FIELD \| CONSTR \| METHOD |  |


---
